# Avoidant/restrictive food intake disorder, other eating difficulties and compromised growth in 72 children: background and associated factors

**DOI:** 10.3389/frcha.2023.1179775

**Published:** 2023-06-20

**Authors:** Maria Johansson, Jonas Hermansson, Petra Linnsand, Christopher Gillberg, Gudrun Nygren

**Affiliations:** ^1^ Gillberg Neuropsychiatry Centre, Sahlgrenska Academy, University of Gothenburg, Gothenburg, Sweden; ^2^ Child and Adolescent Specialist Centre, SV Hospital Group, Gothenburg, Sweden

**Keywords:** ARFID, other eating difficulties, compromised growth, background factors, children

## Abstract

This is a study of avoidant/restrictive food intake disorder (ARFID), other feeding disorders, and background factors, including seventy-two children (thirty-one girls, forty-one boys, aged 4–178 months) referred to a secondary/tertiary feeding service for eating difficulties and/or compromised growth. An in-depth review of their medical records was performed. Diagnostic criteria for ARFID were met in 26% of cases. Children with ARFID were older, more nutritionally deficient, and psychosocially impaired, and their feeding difficulties were less likely to go into remission. Most children's onset of feeding difficulties occurred during the first year of life. Several medical and/or psychosocial and/or neurodevelopmental background factors were often recorded in the same child, regardless of the presence of ARFID or not. Neurodevelopmental disorders were significantly more common in children with ARFID. In conclusion, feeding difficulties in children are often complex, with several associated factors. In a clinical setting, such as the present study, ARFID can be expected in about one-fourth of cases. The feeding difficulties in children with ARFID can be expected to be more severe and persistent than other feeding difficulties. Healthcare providers should be aware of possible underlying neurodevelopmental difficulties in children with ARFID.

## 1. Introduction

“Feeding difficulties” among children is a broad concept which can refer to frequent vomiting, inadequate growth, lack of interest in food and eating, picky eating, and behavioral problems during meals. Feeding difficulties often start between six months and four years of age. A quarter of all children in the general population have been estimated to have some type of mild or severe feeding difficulties during the first years of life, ten percent such that a diagnostic label is appropriate (1).

The genesis of feeding difficulties is complex. All medical disorders, food allergies, congenital malformations and syndromes, chronic diseases affecting the heart, intestines, lungs, brain and kidneys, chronic inflammatory diseases and infections, especially repeated, frequent infections, can affect feeding and growth in children (2, 3). Psychological factors in the context of the caregiver-child dyad also affect children's feeding and growth. Feeding difficulties are associated with both permissive and authoritarian parenting style (4). Anxiety, depression, stress, and behavioral features of eating disorders in mothers can affect behaviors in the feeding situation (5, 6). Maternal depression is related to slow weight gain in infants (7). Research has shown that the prevalence of underweight is higher in children and adolescents in ethnic minority groups, families with low economic standards and homes with a different primary language than the primary language in the country (8, 9). According to Manikam and Perman (10), developmental, psychological, and environmental causes interact to give rise to and maintain feeding disorders, which should be conceptualized as a continuum between organic and psychosocial factors.

Feeding difficulties are common in children with autism, with prevalence rates estimated at 50% up to 90% (11). Feeding difficulties in autism are often manifested early (12) and have been described to mainly fall into three categories (1): selective eating—reported to be the most common type (2), food refusal, and (3) problematic behavior during meals (13–15). The literature has been limited regarding a possible link between feeding disorders and other neurodevelopmental disorders (NDDs). Nevertheless, Hölcke et al. (16) and Råstam et al. (17) indicated an association in children between feeding disorders and attention-deficit/hyperactivity disorder (ADHD).

Current diagnostic classification categories for NDDs in the 5th Edition of the Diagnostic and Statistical Manual of Mental Disorders (DSM-5) (18) and the International Classification of Diseases (ICD-11) (19) only describe behavioral symptoms, and so other difficulties, such as feeding difficulties, in children with NDDs, have often been overlooked. On the contrary, the ESSENCE concept, launched by Gillberg (20), emphasizes the overlap between different behavioral and cognitive problems, which usually onset before three years of age and include, in addition to diagnoses such as autism and ADHD, motor and language difficulties, learning difficulties, sleep problems and feeding difficulties. It also includes excessive crying and problems with eating and sleep in early infancy, often referred to as regulatory problems (21).

Avoidant/restrictive food intake disorder (ARFID) was introduced in the DSM-5 as a new diagnostic term for children whose feeding disorders did not fit the DSM-IV diagnostic categories (18, 22, 23). ARFID has also been included in the ICD-11 (19). The ARFID diagnosis can be applied to children, adolescents, and adults with inadequate nutritional intake due to restrictive eating, which is not due to disturbed body image. According to DSM-5 criteria, ARFID is manifested as a persistent failure to meet adequate nutritional or energy needs associated with significant weight loss and/or significant nutritional deficiencies and/or dependence on enteral or oral nutrition and/or marked effects on psychosocial function (24). There are data suggesting that ARFID includes three often overlapping presentations: (1) lack of interest in eating, (2) restrictive eating due to sensory sensitivity, and (3) avoidance of eating because of fear of negative consequences (25, 26).

To our knowledge, no population-based prevalence estimates on ARFID based on in-depth clinical assessment are yet available. Some population-based studies, relying on self- or parent reports, have shown a prevalence in children, mostly ranging from 0.3%–5.5% (27–31), with only one study reporting a prevalence as high as 15.5% (32). Bertrand et al. (33) estimated the prevalence of ARFID in children aged 0–18 years consulting general pediatricians to 3% (after adjustment based on the French population by age group). A surveillance study by Canadian pediatricians found an incidence of ARFID of 2.02 per 100,000 children aged 5–18 years (34). Prevalence estimates from clinical feeding/eating disorder populations range from 8.4 to 64% (35–45) and have been based on populations differing in several respects, such as patients' age and type of healthcare organization.

Several authors have put forward that ARFID and other neurodevelopmental and psychiatric disorders, particularly autism, ADHD and anxiety disorders, may be overlapping (36, 39, 42, 46–50). Autism spectrum disorder has been reported in 6.1% to 54.8% of children and adolescents with ARFID (34, 36, 44, 45, 47, 51–53).

Several studies also describe medical conditions/symptoms in pediatric clinical settings of patients with ARFID. Fischer et al. (43) found comorbid medical conditions in 50%, gastrointestinal symptoms in 19.4% and food allergies in 4.15% of children with ARIFD, 8–18 years old. Lieberman et al. (42) reported a history of abdominal pain in 31.0%, infections in 37.5%, and food allergies in 13.8%, preceding the ARFID diagnosis in children aged 8–13 years. Krom et al. (40) reported medical diseases in almost 90% of children aged 0–10 years with ARFID. Katzman et al. (34) found medical signs or symptoms, most of whom recognized as consequences of malnutrition, most commonly constipation, in 44.9% of children aged 5–18 years.

In summary, though substantial research has been done on ARFID since the concept was introduced ten years ago, many questions remain about prevalence, clinical characteristics and etiology, and how the diagnostic criteria may best be described.

The Child and Adolescent Specialist Centre (CASC) is a specialist clinic at Angered Hospital in the northeastern part of Gothenburg, Sweden. This area has a high prevalence of inhabitants with low socio-economic standard, and they are more often affected by diseases related to negative lifestyle factors than people in the rest of Gothenburg and Sweden. In 2018 (when the children included in this study were assessed at the TFD), 76.2% of the inhabitants were born in a country other than Sweden or had two foreign-born parents (54). CASC provides health care for children and young people aged 0–18 years with several specialized teams, including teams for children with feeding difficulties (TFD) and teams for NDDs. The TFD was formed because many children referred to the clinic for ailments other than feeding difficulties were found to have underweight, although no obvious explanation for the underweight was found, neither underlying physical cause nor lack of food or care. In 2018, children aged 0–18 years with compromised growth and/or eating difficulties, without disturbed body- and weight perception, were accepted for interventions by the team.

The background to this study is that the co-workers in the TFD, who had yet to start to use the ARFID diagnosis when the study was planned, needed to find out how many children treated by the TFD might meet the diagnostic criteria for ARFID. Further, the co-workers in the TFD had the impression that the children in the team often had NDDs. When planning this study, aware of the complexity of feeding disorders, we decided to analyze all potential risk factors and all concomitant conditions and circumstances possibly maintaining or otherwise impacting the feeding difficulties. In the following, the term *background factors* will refer to all factors and disorders possibly causing or impacting the feeding difficulties, thus both possible underlying etiologic factors and all concomitant conditions and circumstances possibly maintaining the feeding difficulties.

## 2. Aims

The present study aimed to describe the following parameters in children with compromised growth and/or eating difficulties:

• the occurrence of feeding difficulties fulfilling DSM-5-criteria for ARFID
• background factors possibly triggering, maintaining, or otherwise having an impact on eating difficulties and/or compromised growth
• differences regarding background factors, type of feeding difficulties and remission rates of feeding difficulties in children with and without ARFID
• degree of overlap between ARFID and NDDs

## 3. Methods

### 3.1. Participants

Tables 1, 2 shows data on demographics, anthropometrics and remittance. The study included seventy-two children assessed at the TFD at Angered Hospital between January 1, 2018, and December 30, 2018. Thirty-one (43.1%) were girls, and forty-one (56.9%) were boys. The average age of the children at their first visit to the TFD was forty months (range 4–178 months). Sixty-five (90.3%) were less than seven years of age. These children will in the following be referred to as preschool children (in Sweden, children start school at seven years of age). Seven children were school-aged (7–15 years of age). Forty-seven children (65.3%) were referred from Child Health Care (CHC). Almost 80% of the children (57/72) lived in the northeastern area of Gothenburg. Compromized growth (*n* = 59) and/or eating difficulties (*n* = 21) were the most common reasons for referral. There was no attrition of participants during the study.

**Table 1:** Background factors as regards demographics, anthropometrics, medical complications and risk factors during pregnancy, birth and neonatal period, and regulatory problems (*n* = 72).

	Total sample (*n* = 72)	ARFID group (A) (*n* = 19)	Non-ARFID group (B) (*n* = 53)	Difference between A and B
	*n (%)*	*n (%)*	*n (%)*	*p*-value
Gender				
Male *n* (%)	41 (56.9)	10 (52.6)	31 (58.5)	0.658
Female *n* (%)	31 (43.1)	9 (47.4)	22 (41.5)	
Mean age in months at first visit to the TFD^a^ (range)	40 (4–178)	58 (10–147)	33 (4–178)	0.029
Gestational age in weeks^b^				
<37 (%)	3 (4.2)	1 (5.3)	2 (4.0)	0.839
37–42 (%)	60 (93.8)	16 (84.2)	44 (88.0)	
>42 (%)	1 (1.4)	0 (0.0)	1 (2.0)	
Small for gestational age (SGA)^c^	4 (5.6)	0 (0)	4 (7.5)	0.218
Mean birth weight^d^ (gram)	3,265	3,220	3,278	0.755
Mean birth length^e^ (cm)	49.6	49	50	0.495
Medical complications during pregnancy^f^ *n* (%)	5 (6.9)	3 (18.8)	2 (5.0)	0.103
Mother smoked during pregnancy^g^ *n* (%)	5 (6.9)	3 (15.8)	2 (3.8)	0.077
Tobacco exposure during neonatal period^h^ *n* (%)	12 (16.7)	5 (26.3)	7 (13.2)	0.243
Birth complications^i^ *n* (%)	11 (15.3)	2 (10.5)	9 (17.0)	0.798
Medical conditions during neonatal period^j^ *n* (%)	5 (6.9)	2 (10.5)	3 (5.7)	0.405
*Sleeping problems and/or excessive crying*	15 (20.8)	10 (52.6)	6 (11.3)	<0.001
Sleeping *n* (%)	5 (8.3)	4 (21.1)	1 (1.9)	
Excessive crying *n* (%)	4 (5.6)	1 (5.2)	3 (5.7)	
Sleeping and excessive crying *n* (%)	6 (8.3)	5 (26.3)	1 (1.9)	

^a^ TFD: Team for Feeding Disorders.

^b^ Data on gestational age available in 64 children.

^c^ >2 SD below the mean birth weight for the gestational age according to Swedish birth weight standards. Information available in 64 children.

^d^ Data on birth weight available in 70 children.

^e^ Data on birth length available in 68 children.

^f^ Such as gestational diabetes, intrauterine growth retardation and preeclampsia. Data on medical complications during pregnancy available in 56 children.

^g^ Mother smoked in the home during pregnancy. Data available in 63 children.

^h^ Mother and/or father smoked in the home during neonatal period. Data available in 63 children.

^i^ Such as elective and emergency caesarean section and assisted birth with vacuum extraction. Data on birth complications available in 65 children.

^j^ Such as neonatal jaundice requiring treatment, neonatal respiratory disorder requiring neonatal ward, extreme prematurity requiring neonatal ward, neonatal infection requiring neonatal ward. Data on medical complications during the neonatal period available in 62 children.

**Table 2:** Background data regarding remittance (*n* = 72).

	Total sample (*n* = 72)	ARFID group (A) (*n* = 19)	Non-ARFID (B) group (*n* = 53)	Difference between A and B
	*n (%)*	*n (%)*	*n (%)*	*p-*value
Referring unit				
CHC^a^	47 (65.3)	7 (36.8)	40 (75.5)	0.018
SHC^b^	5 (6.9)	2 (10.5)	3 (5.7)	
PHC^c^	7 (9.7)	2 (10.5)	5 (9.4)	
CHC + PHC	1 (1.4)	0 (0.0)	1 (1.9)	
Pediatric clinics	8 (11.1)	6 (31.6)	2 (3.8)	
Self-referrals	4 (5.6)	2 (10.5)	2 (3.8)	
Reason for referral				
Compromised growth	43 (59.7)	6 (31,6)	37 (69.8)	0.067
Eating difficulties	11 (15.3)	5 (26.3)	6 (11.3)	
Eating difficulties + compromised growth	7 (9.7)	3 (15.8)	4 (7.5)	
Eating difficulties + compromised growth + something else	1 (1.4)	0 (0)	1 (1.9)	
Compromised growth + something else^d^	8 (11.1)	4 (21.0)	4 (7.5)	
Eating difficulties + something else	2 (2.8)	1 (5.3)	1 (1.9)	

^a^ CHC: Child Health care.

^b^ SHC: School Health care.

^c^ PHC: Primary Health Care.

^d^ Something else in ARFID group; constipation, swallowing difficulties, follow up of prematurity, reintroduction of cow milk, request for neurodevelopmental assessment, delayed language development, deviant contact and delayed motor development.

Something else in non-ARFID group: vitamin D deficiency, iron deficiency anemia, constipation, suspicions of eosinophilic esophagitis, swallowing difficulties, excessive crying and anxiety disorder.

### 3.2. Data collection

The first author (MJ) scrutinized all available medical records with a closing date of March 30, 2021. Medical records from the CHC, School Health Care (SHC) and other clinics for children who had been assessed for other medical concerns, were collected to provide the best possible background for the assessments in this study.

All data describing feeding difficulties and data on the following categories of background factors were collected: demographics (age at onset of feeding difficulties/the first visit to the TFD/termination of contact with the TFD, sex, place of residence), anthropometrics [growth curves including birth weight, birth length and body mass index (BMI)], data regarding remittance (referring unit, the reason for referral), medical complications and risk factors during pregnancy, birth and neonatal period, medical conditions in the child, NDDs and neurodevelopmental symptoms (NDS), psychosocial factors in the family, and other regulatory problems than eating difficulties. Regulatory problems were categorized as, and will in the following be referred to, as a separate entity since it is not an obvious choice to relate them either to medical or neurodevelopmental factors. Poor dental health was grouped under psychosocial background factors since previous research has concluded that social factors such as family income, parental educational level and ethnicity predict poor dental health in children (55, 56). Medical conditions in the child, NDDs, NDS, psychosocial factors in the family and regulatory problems were recorded if documented in medical records at any time after the child's first visit to the TFD until the end of March 2021. Also, the development of such factors over time was recorded.

Data on medical complications during pregnancy, birth and perinatal period, birth weight, birth length, number of children born as small for gestational age (SGA) and tobacco exposure were compared with statistics from the National Medical Birth Register (57) or with research on such data in Sweden during the current period.

The study was approved by the Regional Ethics Review Board in Gothenburg, Sweden, registration number 2019-00273 and 2020-02174. Due to the nature of the study as a retrospective chart review in which informed consent was not obtained from participants, it was not considered ethically justifiable to collect information about ethnicity, socioeconomic circumstances, neurodevelopmental problems, or mental illness in the family. Therefore, in this report, the term psychosocial factors refer to data about breastfeeding, exposure to tobacco during pregnancy and infancy, food and mealtime routines in the home, diet offered at home, parental style and practices at mealtimes, and dental health.

A review form was prepared for data extraction from medical records. It compromized headings of all data in each table included in this report. Medical records were scrutinized, and all data relating to the areas outlined above were noted, picturing the course of the eating difficulties, the child's development and medical history, and psychosocial factors.

### 3.3. Measures

#### 3.3.1. BMI and growth charts

In Sweden, growth charts are used to monitor child growth. They include standard deviation (SD) curves with growth curves for the mean and 1, 2, and 3 SDs below and above the mean. These have been used to describe growth and BMI in the study. The SD curves can also be understood as percentiles with the following distribution: −3 SD: 0.1%, −2 SD: 2.3%, −1 SD: 15.9%, M: 50.0%, 1 SD: 84.1%, 2 SD: 97.7%, and 3 SD: 99.9%.

#### 3.3.2. Medical assessments including laboratory analyses

Analyzed data included physical examination by a pediatrician and data from other medical assessments (dietitian assessment of nutritional status, gastroscopy, otorhinolaryngological examination, colorectal ultrasonographic examination, and dental examination). Some blood tests, including hemoglobin and iron status, transglutaminase IgA antibodies and vitamin D, had been analyzed for almost all children. Allergy tests, including tests for cow milk protein, had been conducted in children aged six months to three years. In a few children, tests for thyroid stimulating hormone and thyroxine, growth factors and growth factor binding proteins, calprotectin, and routine urine tests, had been analyzed.

#### 3.3.3. The ESSENCE-Q and estimation of probability for NDD

The ESSENCE-Q has been developed as a brief screener to identify children with neurodevelopmental difficulties who might have NDDs (20, 58) (Supplementary S1). The questionnaire consists of twelve items covering: general development, motor development, sensory reactions, communication/language, activity or impulsivity, attention/concentration, social interaction, behavior, mood, sleep, feeding, and “funny spells”/absences. Items can be scored as Yes (2 points), Maybe/A little (1 point), or No (0 points). Total scores range from 0 to 24, with higher scores indicating more difficulties. In the current study, the ESSENCE-Q items were rated retrospectively on data from medical records by the first author (MJ). The following categories for various levels of probabilities for NDD based on ESSENCE-Q scores were set for the study: 0–3 probably no indication, 4–5 some indication, 6–9 moderately severe indication, >10 very severe indication.

### 3.4. Operationalizations for the DSM-5 ARFID-criteria

The DSM-5 criteria for ARFID were used in the diagnostic process (18). Operationalizations for the ARFID criteria A1-A4 in the DSM-5 were defined for the study (Supplementary S2). When planning this study, we hypothesized that some participants would have NDDs. However, we feared it would be challenging to determine if psychosocial impairment in such children would be due to feeding difficulties or other aspects of NDDs. Further, the risk of over-diagnosis, based on clinical evidence of over-reporting of psychosocial impairment by caregivers, has been raised before (59). Therefore, we decided that criterion A4 alone would not be sufficient for an ARFID diagnosis. Hence one of the criteria A1–3, as well as criteria B-D, had to be met.

The operationalizations for the DSM-5 criteria for ARFID defined for the study were applied retrospectively based on available data in medical records by the first author (MJ) for each child. In this process, the nature of the feeding difficulties, whether they were compatible with an ARFID diagnosis, were considered at each point between the child's first visit to the TFD through the end of March, 2021.

### 3.5. Definition of onset of feeding difficulties, remission, psychosocial impairment, nutritional deficiency, sleeping problems, excessive crying, and neurodevelopmental symptoms (NDS)

The following definitions were used:

• Onset of feeding difficulties—the child´s age when concern arose among parents and/or health care professionals about the feeding difficulties.
• Remission of ARFID—eating difficulties no longer meeting the operationalizations for the ARFID criteria defined for the study.
• Remission of feeding difficulties others than ARFID—improvement in growth and eating, and the child considered no longer to need treatment related to these issues.
• Psychosocial impairment and nutritional deficiency—impairment and deficiency as outlined in the study criteria for ARFID for both children meeting and not meeting these criteria (Supplementary S2).
• Sleeping problems—difficulty falling asleep and/or frequent awakenings described by parents to cause problems.
• Excessive crying—crying, according to parentś experience more pronounced than excepted for age and posing a problem.
• NDS—either of the three probability levels for NDD based on rating according to the ESSENCE-Q (see above under heading “The ESSENCE-Q and estimation of probability for NDD”).

### 3.6. Validation study

Chronological case reports were made for the 10 cases in which the diagnostic process regarding ARFID was considered particularly difficult. These reports used collected data to illustrate the course of the feeding difficulties and the child's development, medical conditions in the child and psychosocial factors in the family. The case reports were rated by four raters (except for MJ authors CG, GN and PL), all with extensive knowledge in the field of NDDs and the ARFID concept. Cases were rated regarding whether the child's feeding difficulties fulfilled the study criteria for ARFID or not and to which probability level for NDD description of any NDS in the medical records corresponded.

ARFID diagnoses were assigned according to the judgment of the first author (MJ), regardless of the reliability analysis results.

### 3.7. Statistical analyses

The results are presented as either number of children, means, and proportions. Differences between groups were analyzed with chi-square tests or independent samples t-tests depending on the data type. A *p*-value of 0.05 or less was considered significant. All calculations were carried out using SPSS 25 (60).

## 4. Results

### 4.1. ARFID group

(Table 3 shows characteristics of feeding difficulties in ARFID group.) Nineteen children (26.4%), of whom fifteen (78.9%) were less than seven years old at their first visit to the TFD, had feeding problems meeting the criteria for ARFID at some point after their first visit to the TFD through the end of March 2021. These children will be referred to as *the ARFID group* in the following.

**Table 3:** Characteristics of feeding difficulties (*n* = 72).

	Total sample (*n* = 72)	ARFID group (A) (*n* = 19)	Non-ARFID group (B) (*n* = 53)	Difference between A and B
	*n (%)*	*n (%)*	*n (%)*	*p*-value
Age at onset of feeding difficulties				
0–6 months	30 (41.7)	8 (42.1)	22 (41.5)	0.833
6–12 months	34 (47.2)	8 (42.1)	26 (49.1)	
13–24 months	6 (8.3)	2 (10.5)	4 (7.5)	
25–36 months	2 (2.8)	1 (5.3)	1 (1.9)	
Lack of interest in eating or food	22 (30.6)	19 (100)	4 (7.5)	<0.001
Avoidance based on the sensory characteristics of food	8 (11.1)	5 (26,3)	3 (5.7)	<0.001
Concern about aversive consequences of eating	3 (4.2)	2 (10.5)	1 (1.9)	0.106
Nutritional deficiency^a^	20 (27.8)	13 (68.4)	7 (13.2)	<0.001
Nutritional supplements^b^	41 (58.3)	17 (89.5)	25 (47.2)	0.001
Psychosocial impairment	17 (23.6)	11 (57.9)	6 (11.3%)	<0.001
Remission	45 (62.5)	5 (26.3)	40 (75.5)	<0.001
Terminated contact with TFD	48 (66.7)	7 (36.8)	41 (77.4)	<0.001
	*SDS*	*SDS*	*SDS*	
BMI^c^ at first visit to the TDF^d^	−2.0	−1.7	−2.1	>0.05
Lowest BMI^e^	−2.6	−2.7	−2.6	>0.05

^a^ Deviations in laboratory data (iron deficiency, S-Fe < 15 μg and/or low 25(OH)D-Vitamin < 30 nmol) in need of treatment and/or insufficient nutritional intake according to diarized daily logs (according to dietician assessment).

^b^ Dependence on enteral feeding or oral nutritional supplements after dietician assessment ≥ 1 supplement drink (300-400 kcal) per day.

^c^ BMI: Body Mass Index.

^d^ TFD: Team for Feeding Disorders.

^e^ The lowest measured BMI during the child´s life.

The feeding difficulties started during the first year of life in sixteen of these children (84.2%). In the four school children, the feeding difficulties debuted during the first or second year of life. In five children (26.3%), the study criteria for remission of ARFID were met at the study's endpoint.

### 4.2. Non-ARFID group

(Table 3 shows characteristics of feeding difficulties in non-ARFID group.) Fifty-three children (73.6%) were not found to have “ARFID-level” feeding difficulties during their contact period with the TFD. These children will be referred to as *the non-ARFID group*. Fifty of these (68.5%) were less than seven years old at their first visit to the TFD. Four children in this group showed both lack of interest in food or eating and picky eating, typical of ARFID, of whom two were described as avoiding food because of its sensory characteristics. However, they did not have compromised growth as outlined in the study criteria for ARFID. Lack of interest in food or eating and/or picky eating was documented in ten (19%) additional children but only previously [*n* = 3 (5.7%)] or the description of the eating difficulties was too scant and/or contradictory [*n* = 7 (13.2%)].

The onset of the feeding difficulties occurred during the first year in forty-eight children (91%) in this group. The criteria for remission of the feeding difficulties were met in forty children (75.5%).

### 4.3. Comparison of feeding difficulties in ARFID and non-ARFID groups

(For comparison of feeding difficulties in ARFID and non-ARFID group see Table 3.) The ARFID group had significantly more symptoms within the ARFID subdomains “lack of interest in eating or food”, “avoidance based on the sensory characteristics of food,” and “psychosocial impairment” than the non-ARFID group. No significant differences were found for the subdomain “concern about aversive consequences of eating”, age at onset of feeding difficulties, BMI at the first visit to the TFD, or the lowest measured BMI during the child's life. Nutritional deficiency, as measured with laboratory tests and/or dietitian assessment, as well as nutritional supplements, were significantly more common in the ARFID group than in the non-ARFID group. Remission of feeding difficulties occurred significantly more frequently in the non-ARFID group.

### 4.4. Background factors

#### 4.4.1. Referral unit and reason for referral

The comparison regarding referring unit showed a significant difference between the ARFID and non-ARFID groups (Table 2). A higher proportion of children in the non-ARFID group were referred from the CHC. In contrast, more children with ARFID were referred from pediatric clinics, SHC and by self-referrals from parents.

#### 4.4.2. Gender and age at first contact with the TFD

There were more boys than girls in the ARFID and non-ARFID groups, but no significant difference was found for gender. Children with ARFID were significantly older at their first visit to the TFD than children without ARFID (Table 1).

#### 4.4.3. Medical complications during pregnancy, pre- and perinatal period, birth weight, birth length and number of children born as small for gestational age (SGA)

Gestational age, the number of children born as SGA and the prevalence of medical complications during pregnancy and birth were about the same as in the general population in Sweden (Table 1). Mean birth weight and mean birth length were slightly lower than in Sweden as a whole (57, 61, 62). Somewhat more mothers (6.9%) smoked during pregnancy compared with all pregnant women in Sweden in 2018 (4.2%), and 16.7% of the parents (mother and/or father) in our sample smoked in the home during the neonatal period compared with 10% smoking infant parents across Sweden in 2018 (57).

No significant differences between the ARFID and non-ARFID groups were found for medical complications during pregnancy, birth and neonatal period, the number of children classified as SGA, birth weight, birth length, or tobacco exposure during pregnancy or neonatal period.

#### 4.4.4. Regulatory problems

Sleeping problems and/or excessive crying were documented significantly more often in children with ARFID than in the non-ARFID group (Table 1).

#### 4.4.5. Medical background factors

(Medical background factors are shown in Table 4.) In nine children in the non-ARFID group (17.0%), only one medical factor, such as adenoid hypertrophy, constipation, recurrent infections, cow milk protein allergy, eosinophilic esophagitis, and patent ductus arteriosus, was the only possible background factor considered. The feeding difficulties were in remission at the endpoint of the study in all but one (a child with eosinophilic esophagitis) of these children (15.1%). Constipation was the most common medical condition in both groups, albeit significantly more frequent in the ARFID group. In both groups, infections, mostly recurring upper airway tract infections prior to the feeding difficulties, were the second most common medical condition, and adenoid hypertrophy was the third most common. Treatment of adenoid hypertrophy and cow milk protein allergy did not improve the feeding difficulties in any child with ARFID.

**Table 4:** Medical background factors (*n* = 72).

	Total sample *n* = 72	ARFID group (A) *n* = 19	Non-ARFID group (B) *n* = 53	Difference between A and B
	*n* (%)	*n* (%)	*n* (%)	*p*-value
Constipation	34 (47.2)	13 (68.4)	21 (39.6)	0.044
Infections	18 (25.0)	7 (36.8)	11 (20.8)	0.228
Adenoid hypertrophy	9 (12.5)	4 (21.1)	5 (9.4)	0.189
Cow milk protein allergy	8 (11.1)	2 (10.5)	6 (11.3)	0.905
Constitutional underweight	4 (5.6)	0 (0)	4 (7.5)	–*
Eosinophilic esophagitis	2 (2.8)	0 (0)	2 (3.8)	–
Diarrhea of unknown cause	2 (2.8)	0 (0)	2 (3.8)	–
Patent ductus arteriosus	1 (1.4)	0 (0)	1 (1.9)	–
Choanal atresia	1 (1.4)	0 (0)	1 (1.9)	–
Constitutional short stature	1 (1.4)	0 (0)	1 (1.9)	–
Levels of growth factors on the borderline to low	1 (1.4)	0 (0)	1 (1.9)	–
Tooth eruption	1 (1.4)	0 (0)	1 (1.9)	–
Polydipsia	1 (1.4)	0 (0)	1 (1.9)	–
Allergy to inhalation allergens	1 (1.4)	0 (0)	1 (1.9)	–
Early feeling of satiety	1 (1.4)	0 (0)	1 (1.9)	–
Hyperreactivity to sensory input	1 (1.4)	0 (0)	1 (1.9)	–
Infectious asthma	1 (1.4)	1 (5.3)	0 (0)	–
Obstructive bronchitis	1 (1.4)	1 (5.3)	0 (0)	–
Egg allergy	1 (1.4)	1 (5.3)	0 (0)	–

* *P*-value not calculated if *n* < 5.

#### 4.4.6. Neurodevelopmental background factors

Nine children (42.1%) with ARFID and one child in the non-ARFID group (1.9%) had NDDs (Table 5). Four children had more than one NDD. In the five children with one NDD, data in medical records indicated problems in diagnostic domains other than the diagnosis given to the child. Although only one child was diagnosed with autism, symptoms of autism were indicated in all children with other NDDs.

**Table 5:** Children with neurodevelopmental diagnoses (*n* = 9).

Gender	ARFID	Neurodevelopmental diagnosis	Other data on neurodevelopmental difficulties in medical records	Age at first visit to TFD	Age at neuro-develop-mental diagnosis	Age at onset of feeding problems
Male	Yes	Unspecified LD^a^, ADHD^b^ unspecified type	Further assessments for autism, ADHD and LD planned	10 months^c^	24 months	0–5 months
Male	Yes	Autism, LD (receptive, expressive and pragmatic), ADHD unspecified type		20 months	3 years: 2 months	6–12 months
Male	Yes	PDD NOS^d^ with autistic traits, unspecified ID	Delayed language development, impaired social interaction and eye contact, no play with other children, temper tantrums	2 years: 11 months	4 years: 2 months	6–12 months
Female	Yes	LD (receptive and expressive)	Autism strongly suspected	3 years: 2 months	6 years: 7 months	0–5 months
Female	No	Unspecified LD	Marked delay in language development, stereotypies, echolalia, impaired social interaction and eye contact, parents declined neuropsychiatric evaluation	3 years: 3 months,	3 years: 7 months,	25–36 months
Male	Yes	Phonological LD	Autistic traits, problems with concentration and perception, delayed general language development, reassessment discussed with parents	4 years: 2 months	6 years: 0 months	6–12 months
Female	Yes	ADHD combined type, unspecified ID	Reassessment when 12 years: 10 months showed several autistic traits but criteria for autism were not fully met	10 years: 1 month	9 years: 3 months	13–24 months
Male	Yes	Mild ID^e^	High activity level and sensitivity to sound, strong adherence to daily routines, not socializing with peers, deviant eye contact, no responsive social smile	10 years^f^: 6 months	9 years: months	0–5 months
Female	Yes	ADHD combined type and autistic traits		12 years: 5 months	12 years: 1 month	13–24 months

^a^ LD: Language Disorder.

^b^ ADHD: Attention Deficit Hyperactivity Disorder.

^c^ Mths: months.

^d^ PDD NOS: Pervasive Developmental Disorder Not Otherwise Specified.

^e^ ID. Intellectual Disability.

^f^ Yrs: years.

NDDs and NDS were significantly more common in children with ARFID [*n* = 16 (84.2%)] than in the non-ARFID group [*n* = 11 (20.8%)] (Table 6). The more NDS, the more likely it was that the children were assigned an ARFID diagnosis (*p* < 0.001) (Table 7). Significant differences between the ARFID and non-ARFID groups, with higher scores in the ARFID group, were shown for all ESSENCE-Q items except the item dealing with “Funny spells/absences” (Table 8). The difference between groups was most pronounced for items dealing with sensory reactions, communication, social interaction, behavior, mood, and sleep.

**Table 6:** Medical, psychosocial and neurodevelopmental background factors (*n* = 72).

	Total sample *n* = 72	ARFID group (A) *n* = 19	Non-ARFID group (B) *n* = 53	Difference between A and B
	*n* (%)	*n* (%)	*n* (%)	*p*-value
Medical only or in combination with psychosocial and/or NDDs^a^/NDS^b^	61 (84.7)	17 (89.5)	44 (83.0)	0.502
Medical only	16 (22.2)	1 (5.3)	15 (28.3)	0.038
NDDs and NDS in combination with medical and/or psychosocial	27 (37.5)	16 (84.2)	11 (20.8)	<0.001
NDDs or NDS only	0 (0)	0 (0)	0 (0)	–
Psychosocial only or in combination with medical and/or NDDs/NDS	44 (61.1)	12 (63.2)	32 (60.4)	0.831
Psychosocial only	7 (9.7)	0 (0)	7 (13.2)	0.095
Unknown^c^	1 (1.4)	0 (0)	1 (1.9)	–*

^a^ NDDs: Neurodevelopmental Disorders.

^b^ NDS: Neurodevelopmental Symptoms defined as probability levels for neurodevelopmental disorders as measured with the ESSENCE-Q (ESSENCE-Q scores 4–5: some indication, 6-9: moderately severe indication, >10: very severe indication).

^c^ Feeding difficulties in remission at the time for the first visit to the TFD, why background factors were not considered at assessment, data in medical records did not indicate restrictive or selective eating based on sensory characteristics of food as in ARFID.

* *p*-value not calculated if *n* < 5.

**Table 7:** Neurodevelopmental diagnoses and probability of NDD as measured with the ESSENCE-Q (*n* = 72).

	Neuro-develop-mental diagnosis	Very severe indication of NDD	Moderately severe indication of NDD	Some indication of NDD	Probably no indication of NDD	Total
	ESSENCE-Q: 10–18	ESSENCE-Q: >10	ESSENCE-Q: 6–9	ESSENCE-Q: 4–5	ESSENCE-Q: 0–3	
	(%)	(%)	(%)	(%)	(%)	*n* (%)
ARFID	8 (42.1)	3 (15.8)	1 (5.3)	4 (21.1)	3 (15.8)	19 (26.4)
Not ARFID	1 (1.9)	4 (7.5)	2 (3.8)	4 (7.5)	42 (79.2)	53 (73.6)
Total	9 (12.5)	7 (9.7)	3 (4.2)	8 (11.1)	45 (62.5)	72 (100)

**Table 8:** Symptoms within ESSENCE-Q domains (*n* = 72).

	ARFID group (A) (*n* = 19)	Non-ARFID group (B) (*n* = 53)	Difference between A and B
	*n (%)*	*n (%)*	*p*-value
ESSEENCE-Q total score	10	4	<0.05
General development			
Yes	5 (26.3)	2 (3.8)	0.017
Maybe/A little	1 (5.3)	3 (5.7)	
No	13 (68.4)	48 (90.6)	
Motor development (milestones)			
Yes	3 (15.6)	1 (1.9)	0.036
Maybe/A little	3 (15.8)	4 (7.5)	
No	13 (68.4)	48 (90.6)	
Sensory reactions (e.g. touch, sound, light, smell, taste, heat, cold, pain)			
Yes	11 (57.9)	3 (5.7)	<0.001
Maybe/A little	1 (5.3)	2 (3.8)	
No	8 (42.1)	48 (90.6)	
Communication/language/ babble			
Yes	11 (52.6)	4 (7.5)	<0.001
Maybe/A little	1 (5.3)	4 (7.5)	
No	8 (42.1)	45 (84.9)	
Activity (overactivity/passivity) or impulsivity			
Yes	6 (31.6)	4 (7.5)	0.002
Maybe/A little	7 (36.8)	9 (17.0)	
No	6 (31.6)	40 (75.4)	
Attention/concentration/ “listening”			
Yes	5 (26.3)	2 (3.8)	0.011
Maybe/A little	2 (20.5)	3 (5.7)	
No	12 (63.2)	48 (90.6)	
Social interaction/interest in other children			
Yes	5 (26.3)	5 (9.4)	<0.001
Maybe/A little	5 (26.3)	1 (1.9)	
No	9 (47.4)	47 (88.7)	
Behavior (e.g. repetitive, routine insistence)			
Yes	7 (36.8)	3 (5.7)	<0.001
Maybe/A little	3 (15.8)	1 (1.9)	
No	9 (47.4)	49 (92.5)	
Mood (depressed, elated/manic, extreme irritability, crying spells)			
Yes	6 (31.6)	3 (5.7)	<0.001
Maybe/A little	4 (21.1)	1 (1.9)	
No	9 (47.4)	49 (92.5)	
Sleep			
Yes	4 (21.1)	1 (1.9)	<0.001
Maybe/A little	5 (26.3)	1 (1.9)	
No	10 (52.6)	51 (96.2)	
Feeding			
Yes	19 (100.0)	32 (60.4)	0.010
Maybe/A little	0 (0.0)	2 (3.8)	
No	0 (0.0)	19 (35.8)	
“Funny spells”/ absences			
Yes	0 (0.0)	1 (1.9)	0.547
Maybe/A little	0 (0.0)	0 (0.0)	
No	19 (100)	52 (98.1)	

One child with NDD (11.1%) and ten children with NDS (55.6%) were not considered to have eating difficulties fulfilling the diagnostic criteria for ARFID. In seven of these eating difficulties characteristic of ARFID were documented but not compromized growth or nutritional deficiency as outlined in diagnostic criteria for ARFID (*n* = 4), or they had shown a lack of interest in eating and food and/or avoidance based on the sensory characteristics of food before but not anymore (*n* = 2), or the descriptions of eating difficulties in medical records were considered too scant and contradictory (*n* = 1).

#### 4.4.7. Psychosocial background factors

(Psychosocial background factors are shown in Table 9.) Adverse parental feeding styles and practices were significantly more common in the ARFID group than in the non-ARFID group. Coercive parenting feeding style and distraction during meals were recorded in both the ARFID and non-ARFID groups, whereas indulgent parenting feeding style only was documented in three children in the non-ARFID group. Adverse parental feeding styles and practices were always, except for in one child in the non-ARFID group, recorded together with medical background factors and/or NDDs/NDS. The feeding difficulties were not in remission in any child with ARFID (0/7) and four out of five children in the non-ARFID group, for which adverse parental feeding styles and practices were documented.

**Table 9:** Psychosocial background factors (*n* = 72).

	Total sample *n* = 72	ARFID group (A) *n* = 19	Non-ARFID group (B) *n* = 53	Difference between A and B
	*n* (%)	*n* (%)	*n* (%)	*p*-value
Poor mealtime routines	25 (34.7)	5 (26.3)	20 (37.7)	0.370
Poor diet	18 (25.0)	2 (10.5)	16 (30.2)	0.089
Adverse parental feeding styles/practices	12 (16.7)	7 (36.8)	5 (9.4)	0.006
Adverse breastfeeding	6 (8.3)	1 (5.3)	5 (9.4)	0.573
Poor dental health	2 (2.8)	0 (0)	2 (3.8)	–*
Stress triggered by studies	1 (1.4)	0 (0)	1 (1.9)	–

* *p*-value not calculated if *n* < 5.

#### 4.4.8. Medical, psychosocial, and neurodevelopmental background factors solely and concurrent

Medical and/or psychosocial background factors were documented in medical records for all children in the ARFID group and in all but one child in the non-ARFID group (whose feeding difficulties had gone into remission before contact with the TFD) (Table 5). Medical conditions alone or in combination with psychosocial and/or neurodevelopmental background factors were slightly more frequent in children with ARFID than in the non-ARFID group. Medical background factors solely were significantly more frequent in the non-ARFID group. Psychosocial background factors solely were not documented in any child with ARFID and seven children without ARFID (13.2%). In all these seven children, the feeding difficulties were in remission at the end point of the study. Neurodevelopmental background factors were always documented together with medical and/or psychosocial background factors.

### 4.5. Validation study

The result of the reliability analysis of the assignment of ARFID diagnoses and probability levels for NDS is shown in Supplementary S3 and S4. The four raters agreed upon 80% of the cases regarding ARFID diagnoses and 60% as regards probability levels for NDDs. The disagreement regarding probability levels for NDDs never spanned more than two adjacent probability levels.

## 5. Discussion

### 5.1. ARFID prevalence

Out of the seventy-two children in our sample referred to a pediatric clinic for feeding difficulties, nineteen (26.4%) met the study criteria for ARFID. Since preschool children constituted the vast majority of these, the four previous studies reporting on ARFID prevalence in clinical settings, including preschool children, are closest at hand for comparison. In these studies, the prevalence varies from 8.4%–64%. Three of them referred to children in tertiary eating disorder programs, whereas the study by Inoue et al. (37) included children from primary regional medical centers. Three studies (37, 39, 44) comprised, like our study, both preschool children and adolescents, whereas Krom et al. (40) included children aged 0–10 years. The sample reported by Inoue et al. (37) resembled our sample as regards age and care level. However, almost three-quarters of the patients in that study were diagnosed with anorexia nervosa, which was the case also in the study by Cooney et al. (39). To conclude, there are several differences in sample characteristics between existing studies in this field, which makes comparisons in prevalence of ARFID rather difficult.

Most of our sample were preschool children referred from the CHC to the TFD as a secondary care unit. A substantial proportion appeared to have more ordinary feeding difficulties, such as breastfeeding problems and cow milk protein allergy. Other circumstances to consider when comparing the prevalence of ARFID in our study with other reports of ARFID in clinical eating/feeding disorder programs is that eating difficulties *per se* were not mandatory for inclusion in our study (thus permitting children with only poor growth), and the high prevalence of multi-ethnic background and low economic standard in the area in which most families in our sample lived, theoretically having an impact both on the prevalence and character of feeding difficulties. The multi-ethnic background is also of importance when it comes to the interpretation of the children's growth charts. In Gothenburg, growth curves based on longitudinal data from children born in Gothenburg between 1973 and 1975 are used (61). Several studies have put forward that general normative child growth standards are likely to misclassify children with, for instance, Asian ethnicity as having compromised growth and recommended ethnic-specific references (63). Hence, too many children in our sample may have been assigned ARFID diagnoses.

Preschool children constituted the vast majority in our study, and in those, 23.1% met the ARFID criteria, whereas 57.1% of those seven years or older. This finding contrasts with Farag et al. (44), who found ARFID to be more common in the younger patients in their sample and with the ARFID prevalence of 64% recorded by Krom et al. (40) in children aged 0–10 years with a median age (corrected for prematurity) of 1.85 years. The high prevalence of ARFID in the elder children in our study could be explained by the small number of school children (*n* = 7), making chance findings likely. Though, a more likely explanation might be that a larger proportion in the non-ARFID group were toddlers with feeding difficulties typical for this age, whereas the school children had had longstanding feeding difficulties, for which previous interventions had not been successful.

### 5.2. Background factors

A recent review article presented several studies reporting on factors associated with the emergence of atypical feeding behaviors in infants and young children, each report focusing on one or a few factors and concluded that there has been no research synthesis on such factors (64). Despite the prevalence, impact, and complexity of ARFID and other feeding difficulties in childhood, we are not aware of any previous studies trying to map background factors to feeding difficulties as comprehensively as possible, neither in children with ARFID nor in children with feeding difficulties in general.

An overall observation in our study is that a single background factor to the feeding difficulties was found in very few children. It appears that the professionals in the TFD often first had a theory of one causal factor, and if treatment for this was insufficient, another theory replaced or supplemented the first single-cause theory, and if treatment for the second theory was also unsatisfactory, yet another theory was formed. Professionals seem to, in their search for treatable causes of the feeding difficulties, often have identified concomitant conditions and circumstances, for instance, constipation and adverse parental feeding styles and practices, perhaps caused by the feeding difficulties. Once they had arisen, these concomitant conditions and circumstances seem to have contributed to make the feeding difficulties chronic/subchronic. This is not surprising given that both constipation and adverse parental feeding style have been shown to be bi-directionally associated (as a cause to or a consequence of) with feeding difficulties in children (65). Based on previous research (55, 56), poor dental health was recorded as a psychosocial background factor in this study. However, also poor dental health might be bi-directionally associated with feeding difficulties in children. It is well known that children with autism are at increased risk for poor oral health, and one could assume that the same goes for children with feeding difficulties in general (66).

Feeding difficulties not attributable to a concurrent medical condition and not better explained by lack of available food or an associated culturally sanctioned practice is a diagnostic criterion for ARFID. Thus, one might assume that medical and psychosocial background factors would be less common in children with ARFID than in children with other feeding difficulties. Nevertheless, both medical and psychosocial background factors, especially constipation and adverse parental feeding styles and practices, were found slightly more often in our ARFID group than in the non-ARFID group. Given the small number of children in our sample, we cannot conclude that medical conditions and psychosocial background factors, in general, are more common in children with ARFID than in children with other feeding difficulties. However, it is interesting to note that medical and psychosocial background factors were common in the children with ARFID, perhaps often constituting secondary complications to more severe feeding difficulties. Also, the children with ARFID had more nutrient deficiency and psychosocial impairment, and their feeding difficulties less often went into remission. In line with these findings, another recent study conducted by our group found feeding problems meeting ARFID criteria more severe and longstanding than other feeding difficulties in preschool children with autism (67).

Medical complications during pregnancy and perinatal period were not more frequent in our children than in the general population, except for tobacco exposure, which was slightly more common. Also, birth weight and birth length were slightly lower than in the general population. If parental smoking and low birth weight *per se* have contributed to the development of feeding difficulties in our sample, we can only speculate.

Medical conditions and symptoms in patients with ARFID have been reported previously by many (38–40, 42, 43, 68, 69), and were documented in our sample in almost all children. We are only aware of one previous study, by Krom et al. (40), comprehensively reporting various medical conditions in a pediatric ARFID sample. These children, 0–5 years old, had severe feeding difficulties (many were tube fed) and were patients at a tertiary feeding service. Medical conditions were described in almost 90% of them. Many had congenital malformations and diseases of the digestive, respiratory, and circulatory systems. Although recorded medical conditions in our sample were less frequent and less severe than in the study by Krom et al. (40), our results corroborate previous reports of frequent medical conditions in children with ARFID. Remarkable is that several children in our ARFID group had undergone interventions for medical conditions suspected to have caused the feeding difficulties, such as surgery for hypertrophied adenoid, without success.

We consider the co-variation of ARFID, NDDs and NDS an important finding in our study. The occurrence of several NDDs in our children is in line with previous research reporting an overlap of ARFID with several NDDs (36, 39, 42, 46–50). This might indicate that ARFID is not associated with any particular NDD but occurs as a separate neurodevelopmental category within the ESSENCE concept, overlapping with autism and other NDDs. However, all children in our sample with NDDs were documented to display symptoms of autism. Further, scoring according to the ESSENCE-Q yielded a more significant difference between the ARFID and non-ARFID groups for items measuring symptoms commonly attributed to autism than for items measuring symptoms commonly attributed to ADHD, as well as for items reflecting general development and motor development. This might imply that the association between ARFID and NDDs is mainly mediated through autism.

The overlap of sensory sensitivities between autism and ARFID has been taken to provide a common pathway for the comorbidity of autism and ARFID (70). It has also been speculated that other core symptoms in autism, mainly restricted and repetitive behavior may explain the high prevalence of selective eating and ARFID in autism (70, 71). Koomar et al. (70), found deficits in communication ability, motor coordination and adaptive behaviors to be associated with ARFID, although with clearly weaker association than sensory sensitivity and restricted and repetitive behavior. The children in our ARFID group scored significantly higher on ESSENCE-Q items dealing with sensory reactions, communication, social interaction, and overall behavior than children in the non-ARFID group. This may indicate that all behaviors typical of autism, not just specific symptoms, are associated with ARFID.

Psychosocial background factors were documented with about the same frequency in children with and without ARFID. One explanation might be the low socioeconomic status in the area where most families in our sample lived. However, lack of mealtime structure, uniform diet and non-responsive parental feeding practices have often been reported in families with children suffering from feeding difficulties, both because of parentś concern about insufficient food intake or impaired growth (65, 72), and as a cause to greater selectivity and less food consumption (73, 74). It is beyond the scope of this study to draw conclusions about the genesis of the feeding difficulties in our sample. Nevertheless, psychosocial background factors alone were found in seven children in the non-ARFID group, in all of which the feeding difficulties went into remission, and in no child with ARFID. This might indicate that psychosocial background factors more often were the cause of the feeding difficulties in the non-ARFID group. Brigham et al. (75) discussed whether environmental factors such as family meal milieu, availability of fruits and vegetables in the local environment, and exposure to models of healthy eating and/or diverse foods play a role in the pathogenesis of ARFID. Though, to the best of our knowledge, psychosocial factors have seldom been acknowledged in the ARFID literature as neither possible causal nor possible sustaining factors to the feeding difficulties. This might be because ARFID criteria emphasize eating behaviours and that the eating disturbance shall not be attributable to lack of food or culturally sanctioned practices. It could be that this exclusion criterion diminishes the role of psychosocial factors, of whom some culturally dependent (such as knowledge of and access to nutritious food in the home, and parental style and practices at mealtimes) in the genesis of ARFID. Maternal feeding style have been shown to differ in mothers with different ethnicity (76), and consumer food and beverage purchases to differ between different ethnicity groups (77). Nevertheless, psychosocial therapies, focused on changing eating behavior through parental management are often used in the treatment of ARFID (78).

### 5.3. Difficulties in the diagnostic process

The present study was not designed to differentiate between causative, concomitant and “longitudinally underpinning” factors when reviewing the medical records. Therefore, specific measures were taken to improve the quality of assigning ARFID diagnoses and probability levels for NDDs. Firstly, medical records were scrutinized using a particular review form to show the lifetime course of feeding difficulties, neurodevelopmental problems, and other background factors. Secondly, detailed diagnostic ARFID criteria clarifying the defining features of criteria A1-A4 (although criterion A4 alone not was sufficient for an ARFID diagnosis in the present study). Thirdly, case reports were produced for reliability in the assignment of ARFID diagnoses and probability levels for NDDs. The diagnostic ARFID criteria set up for the study helped decide whether impaired growth, nutritional deficiency, and interference with psychosocial functioning should be considered significant. However, in several children, it was difficult to decide whether the feeding difficulties were compatible with the two ARFID subtypes characterized by a lack of interest in eating and restrictive eating due to sensory hypersensitivity. Sometimes, this was due to scant information about eating difficulties. Sometimes the eating difficulties within the two subtypes appeared to constitute a continuum ranging from mild to severe rather than delineated entities and not seldom appeared to change in severity over time. Another difficulty was determining whether the exclusion criteria B and C were met.

We do not believe that our difficulties in the diagnostics of ARFID are due only to shortcomings attributable to retrospective chart reviews, in which available data is limited to what is documented in medical records. We also believe it reflects the complexity of feeding difficulties and validity problems with the heterogenous ARFID diagnostic construct. Such validity problems have been pointed out previously (59, 79, 80). Strand et al. (80) assumed them derived from a weak emphasis on the three ARFID subdomains and weak demarcation towards other disorders. Harshman et al. (79) argued that DSM-5 provides little text guidance on how to operationalize criteria A1-A4, and that future iterations of DSM should provide a clear definition of food avoidance and restriction along with recommendations for ascertainment. Eddy et al. (59) emphasized that researchers, in the diagnostics of ARFID, shall consider developmental stage and context of feeding or eating disturbance (e.g., birth history, medical complications, caretaker feeding dynamics, level of physical skills/functioning) and clarify definitions used in published papers to enable comparability across studies. In the present study, we have tried to follow the guidelines by Eddy et al. (59). Despite the challenges mentioned above, consensus in the diagnostic process of assigning ARFID diagnoses, based on recorded data, was indicated in the reliability analysis. Given that NDS were not recognized systematically during clinical work, our findings regarding probability levels for NDDs ought to be an underestimation rather than an overestimation. However, also for the estimation of probability levels for NDDs, based on recorded data, good inter-rater reliability was shown in the validation study.

### 5.4. Limitations

This study has several limitations. The sample included only seventy-two children in a clinical setting, of whom nineteen had ARFID. All data were collected from medical records. Thus, it would not be possible to draw firm conclusions based on our results that apply to the general population. Nevertheless, our results are possibly quite typical of children referred to a secondary pediatric feeding service. Also, our population of children is possibly more like the general population than in many previous pediatric ARFID studies based on samples of children in tertiary feeding services or specialized care units (including gastroenterological services). We are only in the beginning of understanding factors that may- or may not be associated with the risk of developing ARFID. In this study, we are not addressing that in a more analytical approach than what we describe in our aim, as we are not certain about what factors that are on the causal chain leading to ARFID and what factors that are acting as confounder. The proxies for NDDs in the present study should not be taken to reflect “real clinical NDD status” perfectly. However, given the expertise of the clinicians involved in monitoring and interpreting the data, they are likely to reflect real clinical phenomena. The finding of several important background factors in many children in our study is probably partly a result of difficulties in differentiating between causative, concomitant and “longitudinally underpinning” factors pertaining to retrospective chart reviews. However, we do not think this is the whole explanation but that this difficulty also can be taken to reflect the complexity of feeding difficulties in children, usually affected by both organic and psychosocial factors embodied in the child-caregiver dyad.

### 5.5. Implications

Professionals assessing children with feeding difficulties should be aware that these children very often have other conditions/complicating factors that need to be addressed and that children with ARFID, in particular, have an elevated risk of coexisting neurodevelopmental difficulties.

## Data Availability

The raw data supporting the conclusions of this article will be made available by the authors, without undue reservation.
