# Supplementary material for: Avoidant/restrictive food intake disorder, other eating difficulties and compromised growth in 72 children: background and associated factors
Source: Front Child Adolesc Psychiatry. 2023 Jun 20;2:1179775. doi: 10.3389/frcha.2023.1179775 (PMC11732122; doi:10.3389/frcha.2023.1179775)
Supplement: Supplementary file 1 [file Datasheet1.pdf]

ESSENCE-Q-REV (Gillberg C 2012)

Name of child:	

Age:		Completed by:	
Sex:		Date:	

Please take a few minutes to read and check the following items.

- **Y= Yes**
- **M/AL = Maybe/A little**
- **N= No**

Have you (or anybody else, who?_________________) been concerned for more than a few months regarding child´s

1. General development ☐
2. Motor development/ milestones ☐
3. Sensory reactions (e.g. touch, sound, light, smell, taste, heat, cold, pain) ☐
4. Communication/language/ babble ☐
5. Activity (overactivity/passivity) or impulsivity ☐
6. Attention/concentration/ "listening" ☐
7. Social interaction/interest in other children ☐
8. Behaviour (e.g. repetitive, routine insistence) ☐
9. Mood (depressed, elated/manic, extreme irritability, crying spells) ☐
10. Sleep ☐
11. Feeding ☐
12. "Funny spells"/ absences ☐

If Y or M/AL to any of the above, please elaborate briefly here:
