# Supplementary material for: Avoidant/restrictive food intake disorder, other eating difficulties and compromised growth in 72 children: background and associated factors
Source: Front Child Adolesc Psychiatry. 2023 Jun 20;2:1179775. doi: 10.3389/frcha.2023.1179775 (PMC11732122; doi:10.3389/frcha.2023.1179775)
Supplement: Supplementary file 2 [file Datasheet2.pdf]

Supplement 2

Operationalizations for the DSM-5 ARFID criteria A1-4 used in the present study

Children aged 0-6 years

Criterion A1 - Significant weight loss (or failure to achieve expected weight gain or faltering growth in children): The A1 criterion was defined as BMI ≤ -2SD for more than three months or:

- during the first year of life deflection in height and weight > 1 SD from the individual height and weight curves over three months
- during the second year of life deflection in height and weight > 1SD from the individual height and weight curves over 6 months
- after 2 years of age defection of > 1 SD in combination with negative impact on height growth regardless of time period or defection of > 0.5 SD in weight and length from the individual curves over a year

Criterion A2 - Significant nutritional deficiency: The A2 criterion was defined as deviations in laboratory data (iron deficiency, S-Fe < 15 µg and/or low 25(OH)D-Vitamin < 30 nmol/in need of treatment and/ or insufficient nutritional intake according to diarized daily logs (according to dietician assessment).

Criterion A3 - Dependence on enteral feeding or oral nutritional supplements: The A3 criterion was defined as dependence on enteral feeding or oral nutritional supplements after dietician assessment ≥ 1 supplement drink (300-400 kcal) per day.

Criterion A4 - Marked interference with psychosocial functioning: The A4 criterion was defined as:

- inability to manage mealtimes because of eating difficulties *(always or often problems at meals because the child shows anxiety, aggressive, disruptive, or conflict triggering behaviour at dinner table and/or because the child refuses to eat with others and eats alone)*

and/or

- The child´s eating difficulties causes difficulties and limitations in daily function and social interaction *(always or often problems with meals at preschool and/or social activities with meals because the child cannot eat at preschool and/or avoids social activities with meals or participates but does not eat, or the child cannot eat in preschool or participate in social activities unless special arrangements are made, or marked tiredness or irritability as a result of nutritional deficiency which means that the child cannot, for example, stay in preschool for whole days and/or the child is unable to participate in social activities; social activities include spending time with friends, family, sports etc.)*

Children aged 7-17 years

Criterion A1 - Significant weight loss (or failure to achieve expected weight gain or faltering growth in children): The A1 criterion was defined as BMI ≤ -2SD for more than three months or failure to achieve expected growth with regard to the child/young person`s previous growth curve and available information on parent`s height and onset of puberty, which cannot be explained by another medical condition or unusually late onset of puberty.

Criterion A2 - Significant nutritional deficiency: The A2 criterion was defined as deviations in laboratory data (iron deficiency, S-Fe < 15 µg and/or low 25(OH)D-Vitamin < 30 nmol/in need of treatment and/ or insufficient nutritional intake according to diarized daily logs (according to dietician assessment).

Criterion A3 - Dependence on enteral feeding or oral nutritional supplements: The A3 criterion was defined as dependence on enteral feeding or oral nutritional supplements after dietician assessment ≥ 1 supplement drink (300-400 kcal) per day.

Criterion A4 - Marked interference with psychosocial functioning: The A4 criterion was defined as:

- inability to manage mealtimes because of eating difficulties *(always or often problems at meals because the child/young person shows anxiety, aggressive, disruptive, or conflict triggering behaviour at dinner table and/or because the child/young person refuses to eat with others and eats alone)*

and/or

- The child´s/young person´s eating difficulties causes difficulties and limitations in daily function and social interaction *(always or often problems with meals at school and/or social activities with meals because the child/young person cannot eat at school and/or avoids social activities with meals or participates but does not eat, or the child/young person cannot eat in school or participate in social activities unless special arrangements are made, or marked tiredness or irritability as a result of nutritional deficiency which means that the child/young person cannot, for example, stay in school for whole days and/or the child/young person is unable to participate in social activities; social activities include spending time with friends, family, sports etc.)*
