# Supplementary figures and images for: Avoidant/restrictive food intake disorder, other eating difficulties and compromised growth in 72 children: background and associated factors
Source: Front Child Adolesc Psychiatry. 2023 Jun 20;2:1179775. doi: 10.3389/frcha.2023.1179775 (PMC11732122; doi:10.3389/frcha.2023.1179775)

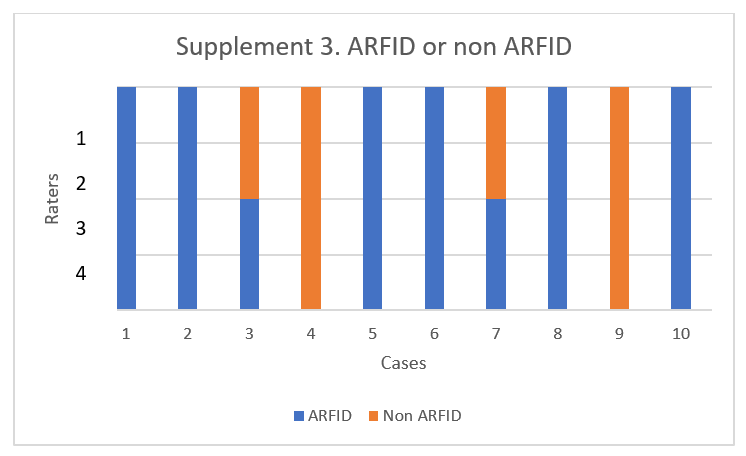

Supplement: Supplementary file 3 [file Image1.png]

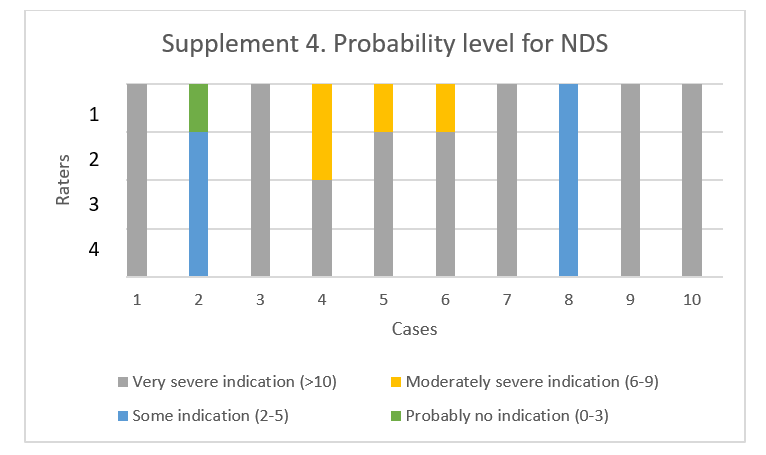

Supplement: Supplementary file 4 [file Image2.png]
